# Parvovirus B19 infection and kidney injury: report of 4 cases and analysis of immunization and viremia in an adult cohort of 100 patients undergoing a kidney biopsy

**DOI:** 10.1186/s12882-020-01911-9

**Published:** 2020-07-09

**Authors:** Maëlis Kauffmann, Mickaël Bobot, Laurent Daniel, Julia Torrents, Yannick Knefati, Olivier Moranne, Stéphane Burtey, Christine Zandotti, Noémie Jourde-Chiche

**Affiliations:** 1 AP-HM, Department of Nephrology, Hopital de la Conception, Marseille, France; 2 Aix-Marseille Univ, C2VN, INSERM 1263, INRA 1260, Campus Timone, Marseille, France; 3 AP-HM, Laboratory of Pathology, Hopital de la Timone, Marseille, France; 4 Department of Nephrology, Hôpital Sainte Musse, Toulon, France; 5 Department of Nephrology, CHU de Nîmes, Nîmes, France; 6 UVE, Aix-Marseille Univ, IRD 190, Inserm 1207, IHU Méditerranée Infection and AP-HM, Marseille, France

**Keywords:** Parvovirus B19, Glomerulonephritis, Thrombotic microangiopathy, Primary infection, Prevalence

## Abstract

**Background:**

The seroprevalence of human Parvovirus B19 (PVB19) is 70–85% in adults worldwide. PVB19 is the etiologic agent of the fifth disease, is a cause of aplastic anemia, and can be associated with kidney injury. We aimed to describe the cases of 4 patients with kidney injury related to PVB19 primary infection, and to evaluate the seroprevalence of PVB19 and the incidence of PVB19 primary infection in patients undergoing a native kidney biopsy.

**Methods:**

Cases of PVB19 infection with kidney injury were reviewed from the archives of the department of Nephrology. A systematic screening of anti-PVB19 IgG and IgM antibodies and viral DNA was performed in sera from 100 consecutive patients with a kidney biopsy in 2017–2018.

**Results:**

The 4 patients with PVB19 infection-associated kidney disease displayed: one lupus-like glomerulonephritis (GN) without lupus auto-antibodies, one minimal change disease with tubular necrosis, one secondary hemolytic and uremic syndrome and one membrano-proliferative GN. In the 100 patients biopsied, 67 had elevated anti-PVB19 IgG, among whom 8 had elevated IgM, without circulating viral DNA, without any particular renal pathological pattern. One additional patient showed a seroconversion at the time of kidney biopsy, which revealed a class V lupus nephritis.

**Conclusion:**

PVB19 primary infection can be associated with different kidney diseases. The seroprevalence of PVB19 among patients with a kidney biopsy is similar to the overall population, and primary infection is rarely documented (1%) after systematic screening. Whether PV19 is nephrotoxic, or triggers renal endothelial injury and immune activation, remains to be elucidated.

## Background

Human parvovirus B19 (PVB19) is a ubiquitous small ssDNA virus, known as the etiologic agent of the fifth disease. Most adults worldwide show evidence of past infection (between 70 and 85%), but a primary infection can occur lately [1]. Infectivity shows seasonal variation, and is more common in spring [2].

In nephrology, PVB19 infection is a matter of concern mainly in kidney transplant recipients, as a cause of aplastic anemia and pure red cell aplasia. The incidence of PVB19 infection after kidney transplantation either as a primary infection or a reactivation, varies between 2 and 30% [3]. PVB19 has also been described as a possible cause of kidney injury. Several cases of glomerulonephritis (GN) occurring after a PVB19 primo-infection have been reported in the literature, although the pathogenic role of PVB19 was difficult to establish. Renal presentation was mostly post-infectious GN, but collapsing focal segmental glomerulosclerosis (FSGS), membrano-proliferative GN, and thrombotic microangiopathy have also been reported [4–8]. Extra-hematological and extra-renal signs can vary from mild or moderate (rash, symmetric arthralgia or arthritis) to severe manifestations (myocarditis, pericarditis, cryoglobulinemic vasculitis, lymphoproliferation), depending on the age, comorbidity, and immunological status of the host [9].

The aims of this study were: 1) to describe the presentations and outcomes of 4 patients who presented with a kidney disease following a primary infection by PVB19 in the department of Nephrology of our University hospital (Hôpital de la Conception, Marseille, France); 2) to evaluate, by a systematic screening, the seroprevalence of PVB19 and the incidence of PVB19 primary infection in a cohort of consecutive patients who underwent a native kidney biopsy in our department.

## Methods

Case reports of kidney diseases occurring after PVB19 infection were gathered retrospectively from the archives of the department of Nephrology, Hôpital de la Conception, AP-HM, Marseille, France.

For the analysis of PVB19 immunization and viremia, samples from 100 unselected consecutive patients who underwent a kidney biopsy in the department of Nephrology between august 2017 and September 2018 were analyzed. All patients gave their written informed consent before any study-related procedure, and samples were included in the biobank DC-2012-1704 (Laboratory of Immunology and Department of Nephrology, Hôpital de la Conception, AP-HM, Marseille, France). The medical history of each patient, and results of blood test with antinuclear antibodies, ANCA, anti MBG, anti PLA2R antibodies and cryoglobulinemia, were reported in the database.

Serum anti-PVB19 IgG and IgM titers were tested by “Liaison °R Biotrin Parvovirus B19 IgG and IgM” kits. PVB19 viremia and the presence of viral DNA in renal tissue was tested by PCR using primers and probe described by Aberham C et al. [10].

Renal pathological examination was performed by two independent renal pathologists (LD and JT). For light microscopy, araldite-embedded sections were stained with Masson’s trichrome and Jones silver impregnation (2 and 0.2 μm sections respectively). For Immunofluorescence 4 μm frozen sections were incubated with anti-Immunoglobulins, C3, and C1q antibodies (The binding site, 1/50 dilutions, Birmingham, UK). For electron microscopy, the biopsy was fixed in 2.5% glutaraldehyde in 0.2 M phosphate buffer, pH 7.4 and then post-fixed in 2% osmium tetroxyde-potassium ferrocyanide. We stained the tissue with 1% uranyl acetate for 3 h. The sample was then dehydrated and embedded in araldite. Ultrathin transverse sections were cut and stained using lead citrate. After staining, these sections were observed under a Jeol 1200 CX electron microscope (Japan).

## Results

### Four cases of PVB19-associated kidney diseases

#### Case report n°1 (2018): lupus-like glomerulonephritis (Fig. 1a)

A 42-year-old women was admitted to the Nephrology department in 2018 for a weight gain of 12 kg over a week, with exertional dyspnea. She had no past medical history. Physical examination revealed normal blood pressure (120/60 mmHg), lower limb edema, and bilateral pleural effusion. A nephrotic syndrome was diagnosed (serum albumin 2.5 g/dL, proteinuria 9.6 g/24 h), with a rise in serum creatinine (1.17 mg/dL), active urinary sediment (red blood cells (RBC) 27/mm3, white blood cells (WBC) 60/mm3), and non-regenerative anemia (hemoglobin 10.7 g/dL, reticulocytes 35 G/L). The complement C3 was low (0.69 g/l), with normal C4 (0.12 g/L), and low CH50 (36%). The exploration of the complement alternative pathway revealed no deficiency in complement factors B, I and H, no antibodies against factor H, and no C3 nephritic factor. No antinuclear or anti-dsDNA antibodies were detected.

**Fig. 1:**
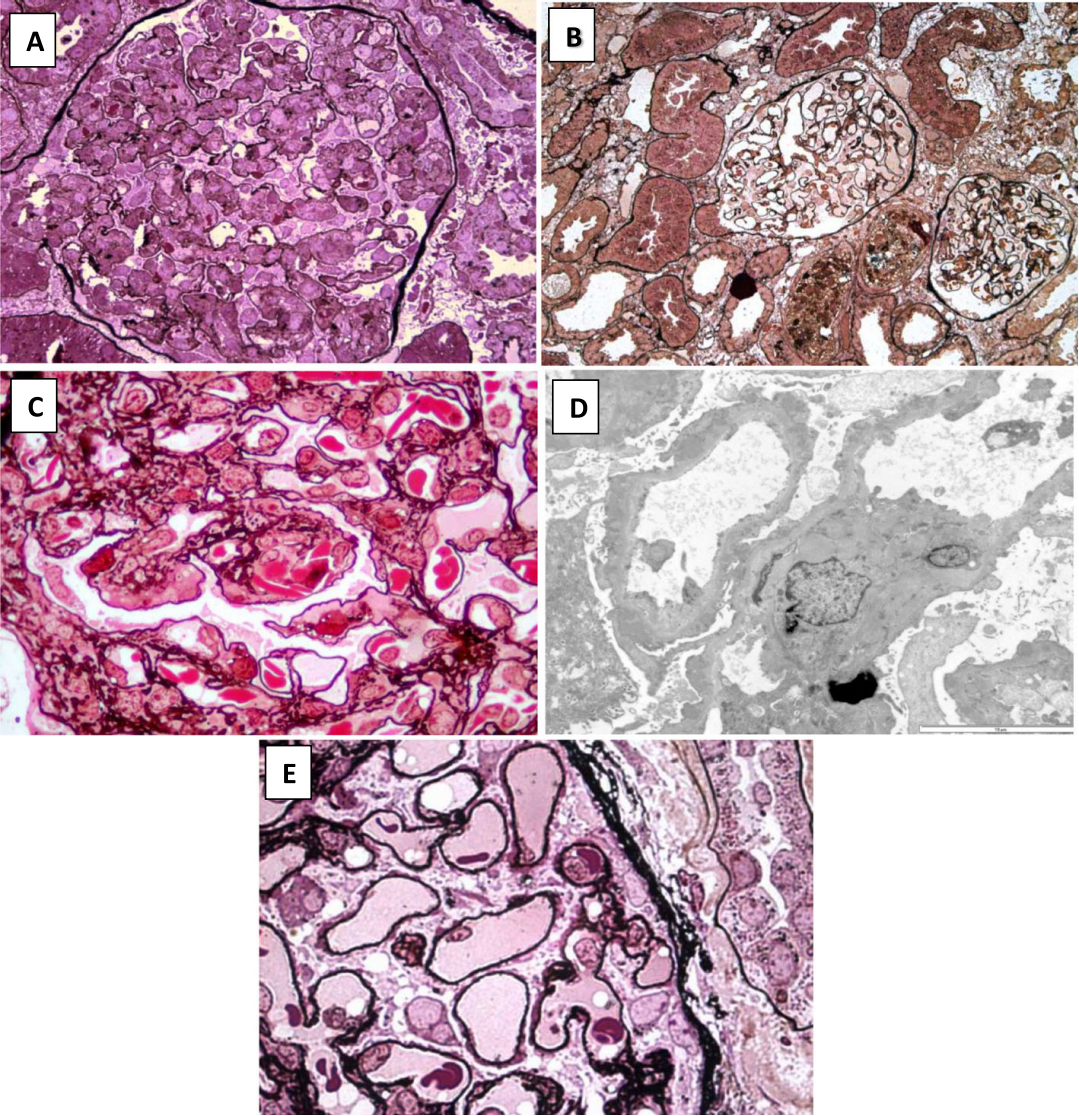
Diversity of renal pathological lesions in patients with primary PVB19 infection. **a** Kidney biopsy of patient 1 showing lupus-like GN. Diffuse and global membrano-proliferative pattern (Jones silver staining, × 400). **b** Kidney biopsy of patient 2 showing minimal glomerular changes and mild alterations of tubular epithelium (Jones silver staining, × 200). **c** Kidney biopsy of patient 4 showing subendothelial deposits without typical duplicated basement membrane and irregular segmental subepithelial deposits. A few sickle cells are seen in glomerular capillaries (Jones silver staining, × 1000). **d** Electron micrograph (× 8000) of patient 4: capillaries show thickening of the basal membrane with many irregular dense granular deposits and diffuse effacement of foot processes. **e** Kidney biopsy of the patient with PVB19 primary infection identified through the systematic screening, showing thickening of the basement membrane with sparse subepithelial deposits (Jones silver staining, × 1000)

A kidney biopsy revealed a membrano-proliferative glomerulonephritis (MPGN), with a “full-house” pattern of endo-membranous deposits comprising IgA (+or-), IgM (++), IgG (+), C3 (++), C1q (+or-).

Anti-PVB19 IgG and IgM antibodies were detected, with a positive viremia, consistent with PVB19 primo-infection. No immunosuppressive therapy was introduced, and because the patient was previously immuno-competent, no intravenous immunoglobulin was administered. A remission of the nephrotic syndrome was observed after 4 weeks, with a decrease in serum creatinine. After 6 months, no relapse has occurred, renal function has normalized (estimated glomerular filtration rate (eGFR) 86 ml/min/1,73 m2) without residual proteinuria (0.09 g/24 h).

#### Case report n°2 (2015): minimal change disease (MCD) and acute tubular necrosis (Fig. 1b)

A 42-year-old women was admitted to the Nephrology department in 2015 for an acute nephritic syndrome. She reported a fever and 3-day rash of the arms and legs 1 month earlier, with a transient consumption of non-steroidal anti-inflammatory drugs. She had no remarkable medical history. Upon admission, she displayed a high blood pressure (190/90 mmHg), with a weight gain of 7 kg over a few days, and gross hematuria. Serum creatinine was elevated (1.33 mg/dL increasing to 2.24 mg/dl), with proteinuria (1 g/24 h rising to 4 g/24 h) and hematuria (> 106/mm3). She displayed non-regenerative anemia (hemoglobin 11 g/dL, which lowered to 7 g/dL before the kidney biopsy, without schizocytes, and a normal haptoglobin level), leukopenia (3.5 G/L), thrombocytopenia (121 G/L), and polyclonal hypergammaglobulinemia. C3 and CH50 levels were low, with a normal C4, without anti-nuclear or anti-dsDNA antibodies. Bone marrow aspiration was normal, and abdominal CT scanner revealed no abnormality.

The kidney biopsy showed minimal change disease and acute tubular necrosis, without immune deposits or glomerular proliferation.

Viral and bacterial serologies were negative (HIV, HBV, HCV, Treponema pallidum), except for PVB19 which showed elevated titers of both IgG and IgM, with a positive viremia. PVB19 was also detected by PCR in the renal tissue.

The patient received oral corticosteroids (initiated at 1 mg/kg/day for 1 month, then decreased over 5 months), and quickly recovered a normal renal function (eGFR 86 mL/min/1.73m^2^), without residual proteinuria (< 0.2 g/24 h). No relapse occurred after steroids withdrawal.

#### Case report n°3 (2012): hemolytic and uremic syndrome (HUS)

A 28-year-old man was admitted to the Nephrology Intensive Care Unit in 2012 for HUS. He reported fever, abdominal pain and non-hemorrhagic diarrhea, 10 days earlier. He came to the emergency room for a worsening asthenia. Physical examination showed only pallor, a blood pressure of 128/97 mmHg, without fever. The blood tests revealed acute renal failure (serum creatinine 6.14 mg/dl), microangiopathic hemolytic anemia (Hemoglobin 11.9 g/dL, undetectable serum haptoglobin, positive schizocytes 1.5%, elevated lactate deshydrogenase 2000 UI/L), without regeneration (reticulocytes 4.2 G/L) associated with a thrombocytopenia (platelets 16 G/L). Urinary tests showed proteinuria (0.8 g/L) and leukocyturia (350/mm3) without hematuria.

Complement fractions were normal, and no abnormality was detected in the complement alternative pathway proteins (normal complement factors B, I, H, absence of antibodies against factor H, absence of C3 nephritic factor). There was no marker of auto-immunity, and the search for E.Coli O157:H7 and Shigatoxin in the stool was negative. Because of the low reticulocyte count, despite hemolytic anemia, a PVB19 infection was suspected and confirmed, with elevated IgG and IgM titers and positive viremia.

One session of plasma exchange was performed, and the patient subsequently reached hematological remission, followed by renal remission. Nine days after his admission, platelet count had normalized (382 G/L), LDH decreased (400 UI/L), and renal function improved (serum creatinine 3.64 mg/dL). No kidney biopsy was performed. The patient recovered a normal renal function (eGFR 95 ml/min/1,73 m2) at 1 month. No relapse occurred subsequently.

#### Case report n°4 (2002): membrano-proliferative glomerulonephritis (MPGN) (Fig. 1c and d)

A 19-year-old woman with a history of sickle cell disease was admitted, in 2002, to the Nephrology department to investigate a nephrotic syndrome. One year earlier, she had presented a PVB19 primary infection which had resulted in an aplastic crisis. Shortly after, she had developed a moderate proteinuria (1 g/day) which had not been explored.

Upon admission, physical examination revealed a normal temperature, blood pressure 130/80 mmHg, pulse 64/min. She was pale, with worsened exertional dyspnea, lower limb edema, and a grade 2/6 systolic murmur. Laboratory tests revealed a nephrotic syndrome (serum albumin 2.4 g/dL, proteinuria 3.5 g/24) with preserved renal function (serum creatinine 1 mg/dL), without hematuria or leukocyturia, and poorly regenerative anemia (hemoglobin 6.3 g/dL, reticulocytes 136 G/L, platelets 882 G/L) without vitamin deficiency. Complement C3 and C4 were normal, and anti-nuclear antibodies were negative. PVB19 viremia was negative, but both IgG and IgM titers remained elevated, rising the hypothesis of a chronic PVB19 infection.

A kidney biopsy was performed and showed a mild endocapillary hypercellularity associated with subendothelial and subepithelial deposits leading to segmental duplicated basement membrane or intervening spikes. Red blood cells within the glomerular capillaries sometimes had a sickled pattern. Immunofluorescence with anti-C3 antibody showed a diffuse, almost continuous, strong staining. Electron micrograph confirmed this thickening of capillary basal membrane with granular dense deposits and effacement of foot processes. Search for PB19 by immunohistochemistry was negative.

The patient received ACE inhibitors, with a remission of the nephrotic syndrome, but with a progressive deterioration of renal function leading to end-stage renal disease 10 years later.

### Prevalence or PVB19 immunization, and incidence of PVB19 primary infection

One hundred consecutive patients who underwent a native kidney biopsy were tested for PVB19 immunization and viremia. Their clinical characteristics and kidney biopsy results are provided in Table 1. There were 41 females and 59 males, with a mean age of 51.5 years; 40 were taking an immunosuppressive therapy (including corticosteroids) at the time of the biopsy.

**Table 1:** Characteristics of the 4 patients with a kidney injury related to Parvovirus B19 primary infection. (NA: not available)

	Case #1	Case #2	Case #3	Case #4
**Gender**	Female	Female	Male	Female
**Age (years)**	42	42	28	19
**Past medical history**	None	None	None	Sickle cell disease
**Clinical examination**	Diffuse edema and pleural effusion	Acute nephritic syndrome, fever, rash	Fever, diarrhea, pallor	Edema, pallor
**Serum creatinine (mg/dL)**	1.17	1.33	6.14	1
**Serum albumin (g/dL)**	2.5	3.3	3.4	2,4
**Proteinuria (g/24 h)**	9.6	1	0.8	3,5
**Hemoglobin (g/dL)**	10.7	8.4	11.9	6,3
**Reticulocytes (G/L)**	35	82	4	136
**C3 level (g/L)**	low	low	normal	normal
**C4 level (g/L)**	normal	normal	normal	normal
**CH50 (%)**	low	low	normal	normal
**Antinuclear antibodies**	negative	negative	NA	negative
**ANCA**	NA	negative	NA	NA
**Cryoglobulinemia**	mixed 2a	mixed 2b	mixed 2	NA
**VHC antibodies**	negative	negative	negative	NA
**PVB19 IgG antibodies**	positive	positive	positive	positive
**PVB19 IgM antibodies**	positive	positive	positive	positive
**PVB19 PCR: blood**	positive	positive	positive	negative
**PVB19 PCR: renal tissue**	NA	positive	NA	negative
**Kidney Biopsy**	Membrano-proliferative GN, full-house pattern	Minimal change disease and acute tubular necrosis	NA	Membrano-proliferative GN, C3 deposits

Elevated titers of anti-PVB19 IgG antibodies were found in 67 (67%) patients, among whom 8 (2 females and 6 males, aged 31 to 83 years) also had elevated IgM titers, without PVB19 viremia (Table 2). The renal pathological results of these 8 patients with both IgG and IgM antibodies were: acute kidney tubular necrosis in 2, minimal change disease in 1, class III lupus nephritis in 1, MPGN with a “full-house” pattern of endo-membranous deposits in 1 (lupus-like GN without lupus auto-antibodies), pauci-immune crescentic GN in 1, hypertensive nephropathy in 1, acute interstitial nephritis with lymphocytic infiltration in 1.

**Table 2:** Characteristics of the 100 consecutive patients who underwent a native kidney biopsy and were tested for Parvovirus B19 immunization

**N**	**100**	**serological profile**			
**Age, mean (range)**	**51.5 (17-88)**	**IgG+ IgM+**	**IgG+ IgM-**	**IgG- IgM+**	**IgG- IgM-**
**Gender, Male/Female (n)**	**59/41**	6 / 2	33/26	0/1	20/12
**Immunosuppressive therapy, yes/no (n)**	**40/60**	2/6	29/30	0/1	9/23*
**Results of kidney biopsy (n)**					
**Primary Glomerulonephritides**					
Minimal Change Disease	**12**	1	6	0	5
Focal Segmental Glomerulosclerosis	**5**	0	4	0	1
IgA Nephropathy	**6**	0	3	0	3
Membranous Nephropathy	**6**	0	3	1	2
Membrano Proliferative Glomerulonephritis	**2**	1	1	0	0
**Secondary Glomerulonephritides**					
Lupus Nephritis	**18**	1	13	0	4
Pauci-immune crescentic glomerulonephritis	**8**	1	6	0	1
**Other Kidney Diseases**					
Hypertensive Nephropathy	**16**	1	10	0	5
Diabetic Kidney Disease	**3**	0	1	0	2
Microangiopathic Hemolytic Anemia	**3**	0	3	0	0
Acute Interstitial Nephritis	**7**	1	3	0	3
Acute Tubular Necrosis	**4**	2	1	0	1
Miscellanous	**10**	0	5	0	5

*p=0.04

One additional patient, a 63-year-old women, initially had elevated anti-PVB19 IgM without IgG antibodies, and subsequently developed anti-PVB19 IgG when she was tested again 4 months later. This was consistent with a PVB19 primary infection at the time of kidney biopsy, although viremia was negative. She had been admitted for arthralgia, and nephrotic syndrome with acute kidney injury (serum creatinine 1.23 mg/dl). Laboratory tests had revealed a non-regenerative anemia (hemoglobin 9,6 g/dl, reticulocytes 26 G/L). Renal pathological examination showed a membranous nephropathy (Fig. 1d). Serum anti-PLA2R antibodies were negative, but there were positive anti-nuclear (> 1/1280) and anti-dsDNA (41 UI/mL) antibodies. The diagnosis retained was a class V lupus nephritis.

Overall, only 1 patient (1%) had a documented primary PVB19 infection in this cohort, and no patient had a PVB19 viremia. Among the 9 patients with elevated IgM titers, 4 had auto-reactive antibodies (antinuclear, ANCA, anti-MBG or PLA2R antibodies), and 6 had a mixed cryoglobulinemia (all with a negative hepatitis C virus serology).

## Discussion

We confirm in this work the rareness of renal involvement during PVB19 infection: the 4 cases identified over 16 years in our center constitutes the largest case series reported in the literature. We report 4 cases of different kidney diseases associated with acute or chronic PVB19 infection, ranging from immune deposition diseases to minimal change disease and thrombotic microangiopathy. The viral infection was suspected, in all cases, upon the observation of an anemia that was either non-regenerative or poorly regenerative, sometimes associated with viral-like symptoms (fever, rash). Although the causality of PVB19 primary infection could not be established in these 4 cases, the timing of kidney injury, the spontaneous favorable course (except in case #4 with chronic PVB19 infection), and the detection viral DNA in the kidney of one patient are in favor of a role of a role of PVB19. The detection of PVB19 DNA in kidney biopsy specimen is indeed often considered as a proof of causality in the kidney injury [4]. Yet, detection of viral DNA in tissues does not necessarily indicate active viral infection and must be interpreted with caution [11].

Only one additional patient with PVB19 primary infection was identified through the systematic screening of 100 consecutive patients who underwent a kidney biopsy. PVB19 infection is thus probably not a frequent and underdiagnosed cause of kidney injury. However, it could be searched for in patients with unexplained lupus-like GN, MCD or TMA, especially after the onset of viral-like symptoms and in periods of seasonal epidemic. Regrettably, as opposed to other viral epidemics, such as flu or gastro-enteritis, no specific health alert is displayed for PVB19 incidence peaks, and PVB19 infection is not a notifiable disease in most countries.

In western countries, PVB19 immunization is observed in 5–15% of children aged 1–5 years, who are the main source of the virus transmission, in 50–60% of older children and young adults, and in over 85% in persons older than 70 years [12–14]. Our results (67% in patients with a mean age of 51 years) are consistent with previous data.

PVB19 viremia, in immunocompetent hosts, occurs 5 to 10 days after exposure and lasts approximately 5 days, with virus titers peaking on the first days of infection. Symptomatic primary infection in adult concerns mostly women (sex ratio 3/1), with a median age of 40 years [15]. By the time symptoms arise, viremia has generally resolved, simultaneously to the seroconversion. PVB19 specific IgM antibodies can be detected from days 10–12 and can persist for up to 5 months. Specific IgG antibodies are detectable at day 15 and usually persist life-long. The development of a robust antibody response corresponds to the virus clearance [16], and no therapy is needed in immunocompetent patients. A chronic infection, with persistent viremia, can occur in immunocompromised patients. Anti-PVB19 antibodies are often undetectable in this setting, and the detection of viral DNA in the blood is the diagnostic gold standard. Treatment with intravenous immunoglobulins has proved to be effective, but no controlled studies have been carried out [17, 18]. Reduction of immunosuppressive medication, when possible, is often recommended.

The specificity and sensitivity of PVB19 specific IgM assay vary between 70 and 100%, meaning that false positive results are possible [19]. Autoimmune antibodies, such as a rheumatoid factor, cryoglobulinemia or antinuclear antibodies, can cross-react with PVB19 serology and yield false positive IgM [20]. Most patients with positive anti-PVB19 IgM antibodies from the present cohort had a mixed cryoglobulinemia. This could either reflect a cause of false positive serological results, or con-firm the suspected role of PVB19 in the development of infectious cryoglobulinemia. Although hepatitis C virus (HCV) infection is the most frequent cause of infectious cryoglobulinemia (70–90%) [21], several case reports of PVB19-related cryoglobulinemic vasculitis have been reported [22, 23]. In the French nationwide CryoVas survey, among 18 patients with non-HCV-related infectious cryoglobulinemic vasculitis, one (5%) was related to PVB19 [24]. A tendency for a higher seroprevalence of PVB19 was also documented among patients with cryoglobulinemia compared to a control group (64.9% versus 50%, NS) [25].

Moreover, a transient positivity of anti-neutrophil cytoplasmic antibodies (ANCA), with anti-proteinase 3 or anti-myeloperoxidase specificity, has been reported in 10% of patients during acute PVB19 infection [26], and is a potential pitfall for the diagnosis of ANCA-associated vasculitis. Recently, an association between persistent PVB19 infection and the production of antiphospholipid antibodies in pediatric and adult patients with rheumatic diseases has also been described [27]. Molecular mimicry could be a major pathogenic mechanism triggering the production of auto-antibodies after PVB19 infection [28]. Several reports have described lupus-like manifestations related to PVB19 infection, including some with acute glomerulonephritis, with transient positive anti-nuclear or anti-dsDNA antibodies [29]. In a murine model, the injection of apoptotic bodies containing dsDNA modified by PVB19 induced the production of anti-dsDNA antibodies and immune-mediated organ damage [30]. Although PVB19 infection may be involved in the pathogenesis of (auto)immune-mediated diseases, as an infectious trigger, the correct diagnosis of PVB19 infection is particularly important in this setting, to avoid an inadequate and potentially harmful immunosuppressive therapy in some patients [29, 31].

Although it is now established that the pathogenicity of PVB19 is not restricted to the erythroid progenitor cells, the mechanisms of PVB19-related renal lesions are still poorly understood. The proposed mechanisms include cytopathic effects on glomerular endothelial cells, glomerular deposition of immune complexes resulting from the infection, or the development of immune disorders after PVB19 infection. The direct infection of glomerular epithelial cells could be the mechanism of kidney injury in cases of FSGS, including collapsing glomerulopathy [6]. Indeed, PVB19 viral DNA was detected in kidney biopsies of patients with non-HIV-related collapsing FSGS, and was precisely located in visceral and parietal epithelial cells. However, viral DNA was also detected in control samples and in other glomerular lesions, which questions the specificity of this finding [32]. In cases of TMA and vasculitis, the direct infection of glomerular endothelial cells has also been proposed as the trigger of endothelial activation and lesion, leading to the subsequent thrombotic and inflammatory response with complement activation [7, 33]. Indeed, the endocytosis of PVB19 by endothelial cells is enhanced by the presence of anti-PVB19 antibodies and their linkage to the C1q receptor [34]. The glomerular deposition of circulating immune complexes, comprising viral antigens and host antiviral antibodies, could also lead to a post-infectious glomerulonephritis, which is the pathological lesion most frequently described in the literature [35]. In addition, PVB19 could be implicated in the activation of complement alternative pathway (CAP), since C3 level is often decreased in PVB19 infection with kidney involvement [36]. Here, the case report n°4, showing MPGN with C3 deposits following a PVB19 infection in a patient with sickle cell disease, is particularly original and illustrates this possible link between PVB19 and CAP activation.

We acknowledge certain limitations to this study. First, this retrospective work did not allow to evaluate the real incidence of kidney injury associated with PVB19 infection. To overcome this limit as much as possible, we demonstrated through a systematic screening that the incidence of PVB19 primo-infection in patients undergoing a kidney biopsy was very low. Second, 2 of the cases were not fully explored for CAP abnormality. Third, because EM examination is not a routine practice in France, it was available for 1 patient only.

## Conclusion

Kidney diseases associated with PVB19 primary infection are diverse and infrequent. Although PVB19 primary infection is rare in adults, we suggest that PVB19 serology could be performed in patients with atypical presentations of TMA or glomerulonephritis, especially if they were preceded or associated with a viral syndrome or non-regenerative anemia, or occurred in an epidemic context. The diagnosis of PVB19 infection in these patients can spare an inadequate and potentially harmful immunosuppressive therapy.

## Data Availability

All data used and analyzed during the current study are available from the corresponding author on reasonable request.
